# SprI/SprR Quorum Sensing System of *Serratia proteamaculans* 94

**DOI:** 10.1155/2019/3865780

**Published:** 2019-12-12

**Authors:** Yulia V. Zaitseva, Olga A. Koksharova, Valentina A. Lipasova, Vladimir A. Plyuta, Ilya V. Demidyuk, Leonid S. Chernin, Inessa A. Khmel

**Affiliations:** ^1^Institute of Molecular Genetics, Russian Academy of Sciences, Kurchatov sq. 2, Moscow 123182, Russia; ^2^Demidov Yaroslavl State University, Sovetskaya Str. 14, Yaroslavl 150003, Russia; ^3^Lomonosov Moscow State University, Belozersky Institute of Physical-Chemical Biology, Leninskie Gory, 1-40, Moscow 119992, Russia; ^4^Department of Plant Pathology and Microbiology, The Robert H. Smith Faculty of Agriculture, Food and Environment, The Hebrew University of Jerusalem, POB 12, Rehovot 76100, Israel

## Abstract

In this study, we investigated the quorum sensing (QS) regulatory system of the psychrotrophic strain *Serratia proteamaculans* 94 isolated from spoiled refrigerated meat. The strain produced several *N*-acyl-*L*-homoserine-lactone (AHL) QS signal molecules, with *N*-(3-oxo-hexanoyl)-*L*-homoserine lactone and *N*-(3-hydroxy-hexanoyl)-*L*-homoserine lactone as two main types. The *sprI* and *sprR* genes encoding an AHL synthase and a receptor regulatory protein, respectively, were cloned and sequenced. Analysis of their nucleotide sequence showed that these genes were transcribed convergently and that their reading frames partly overlapped by 23 bp in the terminal regions. The genes were highly similar to the *luxI*/*luxR-*type QS genes of other Gram-negative bacteria. An *spr*-box (analog of the *lux*-box) was identified upstream of the *sprR* gene and found to be overlapped with the sequence of −10 sequence site in the promoter region of this gene. Inactivation of the *sprI* gene led to the absence of AHL synthesis, chitinolytic activity, and swimming motility; decrease of extracellular proteolytic activity; affected the cellular fatty acid composition; and reduced suppression of the fungal plant pathogen mycelium growth by volatile compounds emitted by strain *S. proteamaculans* 94. The data obtained demonstrated the important role of the QS system in the regulation of cellular processes in *S. proteamaculans* 94.

## 1. Introduction

In recent years, it has become evident that bacteria regulate various physiological processes depending on their population density. This specific type of regulation is called quorum sensing (QS). QS signaling systems include low-molecular-weight molecules of different chemical nature and regulatory proteins that interact with the signaling molecules. With an increase in the population density, the concentration of signaling molecules increases to a definite threshold value, which may lead to an abrupt activation of the transcription of sets of genes in the entire bacterial population. It has been shown that the QS systems play a key role in the regulation of metabolism in bacteria living in different ecological niches and belonging to different taxonomic groups. Transcriptome and proteome analyses have shown that QS systems function as global regulators of gene expression in bacteria. QS signaling molecules (autoinducers) participate in intercellular transmission of information between bacteria belonging to the same and different species, genera, and even families. Due to QS regulation, bacteria are able to coordinately control gene expression in the population [1–3].

The best studied QS systems are the LuxI/LuxR-type systems first detected in *Vibrio fischeri* that function in Gram-negative bacteria with AHLs as the signal molecules. The QS regulation in *V. fischeri* occurs with the participation of two major components, the AHL synthase LuxI (which catalyzes AHL synthesis) and the LuxR protein. The latter forms a complex with AHL that binds to the promoter of the *lux* operon to activate its transcription, which leads to luciferase production and light emission. Most proteins of the LuxR type in different bacteria function as transcriptional activators, e.g., R proteins of *Pseudomonas*, *Agrobacterium*, *Burkholderia*, and *Rhizobium* [1–4]. In other bacteria of the *Enterobacteriaceae* family (e.g., *Pantoea*, *Erwinia*, *Serratia*, and *Yersinia*), another type of QS regulation is observed. In these bacteria, the LuxR-type proteins act as negative regulators, repressing their transcription and the transcription of target genes. Interaction of AHL with the R protein leads to conformational changes in the protein structure, which inhibit its binding to DNA and cause derepression of transcription [5–9].

The present work is devoted to study the QS regulation of *Serratia proteamaculans* 94 and its role in the control of cellular processes. This strain was isolated from meat spoiled in a refrigerator and identified by an analysis of nucleotide sequences of 16S rRNA genes (accession number EU327084.1) [10]. *S. proteamaculans* bacteria are adapted to various ecological niches in nature. They live in soil and in the rhizosphere of plants and are involved in spoilage of milk, dairy products, and meat [10–12]. Since QS systems play an important role in the adaptation of bacteria to different environmental conditions, including food-related species [13–15], one would expect the presence of actively functioning QS systems in *S. proteamaculans* 94. QS systems of this species are poorly studied. The *S. proteamaculans* B5a strain isolated from cold-smoked salmon was shown to cause QS-controlled spoilage of milk and to produce OHHL as the main AHL. The QS genes of the strain, *sprI* and *sprR*, were sequenced. According to proteome analysis, expression of many proteins in the B5a strain was affected by the QS system [11]. We recently investigated the functional role of the *S. proteamaculans* 94 gene *luxS* responsible for the synthesis of AI-2, a signaling molecule that participates in QS regulation in a large number of bacteria. It was shown that knockout of this gene led to an absence of AI-2 synthesis, chitinolytic activity, and swimming motility, suppression of the growth of the fungal plant pathogens *Rhizoctonia solani* and *Helminthosporium sativum* by volatile compounds emitted by *S. proteamaculans* 94 strain, and a decrease in extracellular proteolytic activity but did not affect synthesis of AHLs and lipolytic and hemolytic activities [16].

In this work, we have shown that *S. proteamaculans* 94 produces several types of AHLs, including OHHL and HHHL as the prevalent types. Genes of its QS system, namely, the *sprI* gene encoding an AHL synthase and the *sprR* gene encoding a receptor regulatory protein, were cloned and sequenced, and the organization of these genes was investigated. A *S. proteamaculans* 94 mutant with an inactivated AHL synthase (*sprI*) gene was constructed, and the role of QS in the regulation of several metabolic and physiological processes and properties in *S. proteamaculans* 94, including extracellular proteo- and chitinolytic activities, swimming motility, antifungal activity related to emission of volatile compounds, and cellular fatty acid composition, was demonstrated.

## 2. Materials and Methods

### 2.1. Organisms, Media, and Growth Conditions

The strains and plasmids used in the work are listed in Table 1. Bacterial strains were grown in Luria-Bertani broth (LB) or on solidified (1.5% w/v agar) Luria-Bertani (LA) agar (Sigma-Aldrich Chemie GmbH, Steinheim, Germany) at 30°C. The fungi *R. solani* and *H. sativum* (from the collection of the Institute of Molecular Genetics, Moscow) were grown at 25°C on PDA (Difco) or Czapek agar medium (in g/l of distilled water): saccharose, 30; NaNO_3_, 2; KH_2_PO_4_, 1; MgSO_4_, 0.5; KCl, 0.5; FeSO_4_, 0.01; and agar-agar, 15.

Antibiotic supplements were used at the following concentrations: ampicillin (Ap, 200 *μ*g/ml, “Biochemist,” Russia), kanamycin (Km, 100–200 *μ*g/ml, “Synthesis,” Russia), gentamicin (Gm, 25 or 40 *μ*g/ml, KRKA, Slovenia); rifampicin (Rif, 50 *μ*g/ml), tetracycline (Tc, 20 *μ*g/ml), and chloramphenicol (Cm, 20 *μ*g/ml), from Sigma-Aldrich (USA).

### 2.2. Assay of AHL Production

An assay of AHLs was performed on plates with the violacein-producing reporter strain *Chromobacterium violaceum* CV026 [18] and the *β*-galactosidase-producing reporter strain *Agrobacterium tumefaciens* NTL4/pZLR4 [19].

### 2.3. Quantitative Determination of AHL Production by Liquid Chromatography and Mass Spectrometry (LC-MS/MS)

An overnight *S. proteamaculans* 94 culture was diluted to a starting OD_600_ of 0.15–0.25 (1 − 3 × 10^8^ CFU·ml^−1^), and the cells were grown for 24 h. AHL was extracted by ethyl acetate (EtAc) as described [24]. In brief, cell-free culture supernatants (50 ml) prepared from the liquid cultures of the AHL-producing strains were acidified to pH 2.0 to prevent additional hydrolysis of AHLs and extracted twice with the same volume of EtAc; the extracted organic phases were then pooled. The solvent was evaporated under vacuum, and the resultant EtAc extracts were dissolved in 50 *μ*l of HPLC-grade acetonitrile and stored at −20°C. An LC-MS/MS analysis was performed using an Agilent 120 Rapid Resolution LC system (Agilent Technologies Inc., Santa Clara, CA, USA) at a 0.25 ml/min flow rate. Mobile phase A (aqueous) was based on 0.05% acetic acid in H_2_O (MS grade), and mobile phase B was based on 0.05% acetic acid in acetonitrile (HPLC grade). The percentage of solvents A and B varied from 5 to 75 and 25 to 95%, respectively. The temperature of the HPLC column was maintained at 30°C, and the injection volume was 5 *μ*l. HPLC separations were carried out using a Phenomenex Gemini C18 HPLC column (2 × 150 mm, particle size 3.5 *μ*m). The liquid chromatography system was coupled with an Agilent 6410 triple quad mass selective detector equipped with an electrospray ionization ion source. The mass spectrometer was operated in the positive ionization mode at a capillary voltage of 4000 V, drying gas (nitrogen) temperature of 350°C, flow rate of 10 l/min, and nebulizer pressure of 35 psi, with nitrogen (99.999%) as the collision gas. The LC-MS system was controlled, and data were analyzed using MassHunter software (Agilent Technologies Inc.). Quantitative analysis of homoserine lactones was performed in the multiple reaction monitoring (MRM) mode by the method of standard addition. A set of synthetic AHLs with or without 3-oxo or 3-hydroxy substitutions and with acyl side chain lengths ranging from C4 to C12 at either 1 or 5 *μ*M concentration was used as standards. AHLs were identified and confirmed by comparison of the elution time and MS spectra of their peaks and those of the standards.

For quantification, the mass spectrometer was configured for selected ion monitoring. The ions selected and the respective AHLs were as follows: *m*/*z* 172, BHL; *m*/*z* 200, HHL; *m*/*z* 214, OHHL; *m*/*z* 216, HHHL; *m*/*z* 228, OHL; *m*/*z* 226, HOHL; *m*/*z* 269, ODHL; and *m*/*z* 297, OdDHL. The concentrations of AHLs in EtAc extracts from supernatants of strain *S. proteamaculans* 94 applied to the column are presented in ng/ml and in *μ*M. All AHL standards were purchased from Sigma-Aldrich Israel Ltd (Rehovot, Israel).

### 2.4. DNA Manipulations

Total genomic and plasmid DNA isolation, restriction enzyme digestion of DNA, ligation, agarose gel electrophoresis, PCR, Southern blot hybridization, electroporation, and transformation of *E. coli* cells with plasmid DNA were generally performed according to standard procedures [22]. Biotin-labeled specific probes were obtained with a Biotin DecaLabel DNA Labeling Kit (Fermentas, Life Sciences, Lithuania). Restriction enzymes and T4 DNA ligase were purchased from MBI Fermentas (Vilnius, Lithuania) and used according to the manufacturer's instructions.

PCR reactions were performed in a total volume of 20 *μ*l containing a buffer for Taq DNA polymerase (“Sylex,” Russia), 200 *μ*M concentrations of each of the four deoxynucleoside triphosphates, 10 рМ of each PCR primer (“Syntol,” Russia), and 1 U of Taq DNA polymerase (Institute of Molecular Genetics, Moscow, Russia). Plasmid DNA or boiled cells obtained from freshly grown colonies were used as templates for PCR. The primers used are listed in Table 2. DNA sequencing was performed with an Applied Biosystems 3730 DNA Analyzer using an ABI PRISM® BigDye™ Terminator v. 3.1 set. BLASTN and BLASTX homology searches for DNA sequences were performed using the NCBI server (http://www.ncbi.nlm.nih.gov/BLAST/).

### 2.5. Cloning and Sequencing of the *sprI* and *sprR* Genes

Identification and cloning of the *sprI* and *sprR* genes were performed using PCR amplification with the degenerate primers deg SprI-F and deg SprI-R. The following program for PCR was used: 94°С for 3 min, followed by 30 cycles at 94°С for 20 s, 50°С for 40 s, and 72°С for 40 s. Boiled cells obtained from freshly grown colonies were used as templates for PCR. PCR products were isolated from gels using a Wizard SV Gel and PCR Clean-Up System (Promega) and were sequenced using the deg SprI-F and deg SprI-R primers. The obtained nucleotide sequences were compared with those in the GenBank database. A fragment that contained the complete *sprI* gene and a fragment of the *sprR* gene (83% identity to the respective gene sequences in *S. proteamaculans* B5a, AY040209.1) was obtained.

DNA fragments containing the whole *sprI* and *sprR* genes were determined by Southern blot hybridization with biotin-labeled specific probes and amplified by PCR. Chromosomal DNA was digested with *Bam*HI and *Hind*III. A DNA fragment containing the *sprI* and *sprR* genes of approximately 1700–1750 bp was detected. A mixture of DNA fragments of 1500–2000 bp in size obtained after restriction digestion was ligated into pACYC184 vector DNA digested with *Bam*HI and *Hind*III and then transformed into *E. coli* TG1. The cells were plated on the LA medium supplemented with chloramphenicol (20 *μ*g/ml). Selected clones were tested for the presence of the *sprI* and *sprR* genes by PCR using the primers deg SprI-F and deg SprI-R (the *sprR* gene was located next to the *sprI* gene) and primers for the pACYC184 vector, pACYC-F, and pACYC-R (Table 2). DNA of the recombinant plasmid pACYC184-*sprIR* was isolated using a GeneJET Plasmid Miniprep Kit (Fermentas (Vilnius, Lithuania)). Sequencing of the cloned DNA sequence containing the *sprI* and *sprR* genes was performed using the primers pACYC-F and pACYC-R, which recognized the vector, and the deg SprI-F and deg SprI-R primers. The nucleotide sequence of the *sprI* and *sprR* genes was determined in a fragment of 1660 bp and deposited in GenBank (JX901285).

### 2.6. Gene Replacement Mutagenesis of the *sprI* Gene

For obtaining an *sprI* mutant, the *sprI* gene was cloned using PCR with specific primers to this gene: SprI-F and SprI-R (Table 2). PCR was conducted at 94°С for 2 min followed by 30 cycles at 94°С for 20 s, 58°С for 20 s, and 72°С for 40 s, and the last step was 72°С for 4 min. PCR products were isolated from gels using a Wizard SV Gel kit and PCR Clean-Up System (Promega, USA) and ligated to pAL-TA (Evrogen, Russia) vector DNA. The ligation mixtures were transformed into *E. coli* TG1. The cells were plated on the LA medium supplemented with 200 *μ*g/ml ampicillin, and the presence of the *sprI* gene in selected clones was tested by PCR using the universal M13 primers. The plasmids obtained were named pAL-TA-*sprI*.

The *sprI* gene was inactivated by inserting a suicide vector into the *S. proteamaculans* 94 chromosome by homologous recombination using the *sacB*-based strategy [21, 25]. To inactivate the *sprI* gene, an 847-bp *Kpn*I-fragment of the p34S-Gm plasmid bearing the gentamicin resistance gene [20] was cloned into the *Kpn*I site of the *sprI* gene in the pAL-TA-*sprI* plasmid. Then, a 1.6-kb *EcoR*I fragment of the obtained plasmid pAL-TA-*sprI*::Gm was cloned into the *Eco*RI site of the pEX18Tc vector. The resulting plasmid pEX18Tc-*sprI*::Gm was used to transform the *E. coli* strain S17-1 (*λ*-*pir*). The transformed cells were in turn used as a donor for plasmid transfer into *S. proteamaculans* 94 by conjugation with selection for Gm-r, Rif-r, and Tc-r transconjugants (the *S. proteamaculans* 94 *rif*-*r* mutant used in this work was obtained previously [16], as a spontaneous mutant selected on the LA medium supplemented with 100 *μ*g/ml rifampicin). The obtained clones were grown overnight in LB with Gm and Rif and then plated on the LA medium supplemented with Gm, Rif, and 5% (w/v) sucrose. The gentamicin-resistant, rifampicin-resistant, sucrose-resistant, and tetracycline-sensitive clones were tested by PCR with the Gm-F and Gm-R primers as well as with the SprI-F and SprI-R primers. As a result, the mutant strain *S. proteamaculans sprI*::Gm was obtained.

### 2.7. Assays of Proteolytic, Lipolytic, Chitinolytic, and Hemolytic Activities

For enzymatic activity assays, 3 *μ*l of a 300 times diluted overnight culture of bacteria was plated on the surface of the solidified medium. The experiments for all enzyme assays were performed as described [26] and repeated in triplicate.

The activity of extracellular proteases was assayed after incubation at 30°C for 48 h on plates with the LA medium supplemented with 0.5% milk (1/3 vol.) by the appearance of casein degradation halos around bacterial colonies. To determine the lipase activity, strains were cultured for 4 days on LA plates supplemented with 0.01% CaCl_2_ and 1% Tween 20. Lipases hydrolyze Tween 20 to produce lauric acid that is converted into its insoluble calcium salt, which leads to an appearance of turbid zones around bacterial colonies. Enzyme activity was estimated by the radius of the turbid zones. The hemolytic activity of bacteria was determined on the LA medium supplemented with 5% sterile blood (human blood from Blood Transfusion Center, Moscow). Hemolytic activity was estimated by the radius of clear zones of hemolysis around bacterial colonies after growth for 6 days at 30°С. To determine the chitinolytic activity, cells were plated on the solidified (1.5% agar) medium containing 0.1% (NH_4_)_2_SO_4_, 0.03% MgSO_4_^.^7H_2_O, 0.08% KH_2_PO_4_, 0.04% KNO_3_, and 0.05% of yeast extract (Difco) supplemented with 0.2% colloid chitin. Bacteria were incubated for 96 h at 30°С. Chitinolytic activity was detected by the appearance of clear zones around bacterial colonies.

### 2.8. Action of Volatile Compounds Synthesized by *S. proteamaculans* 94 on Phytopathogenic Fungi

The antifungal activity experiments were performed in two-compartment Petri plates (92 × 16 mm) filled with the LA medium on the one side and solid Czapek's medium on the other side as described [27]. LA was seeded with the *S. proteamaculans* 94 strain or *sprI* mutant (20 *μ*l of overnight culture, ∼3–5 × 10^7^ CFU, was distributed on the surface of LA) and incubated for 2 h at 28°C. Then, a block of solid Czapek's medium (8 mm in diameter) covered with 5-day-old fungal mycelium was excised and placed onto the Czapek-filled part of the plate. All plates were 4-times sealed with «Parafilm M» Sealing Film (Pechiney Plastic Packaging Company, Chicago, IL, USA) and incubated for 6 days at 25°C.

### 2.9. Swimming Assay

The swimming motility of *S. proteamaculans* 94 cells was measured as described [28], with slight modifications. Overnight cultures grown in LB in tubes at 30°C with shaking were 300-fold diluted with LB and grown for 2.5 h. Then, 2 *μ*l of the culture was plated on the solidified (0.3% Difco agar) M9 medium containing 0.4% glucose and 0.5% casamino acids (Difco), and the diameters of swimming zones were measured after 36 h of growth at 30°C.

### 2.10. Fatty Acid Assay

An overnight *S. proteamaculans* 94 culture was diluted to a starting OD_600_ of ∼0.10, and the cells were grown for 20 h at 30°C. Extraction of fatty acids from dried samples (5 mg) of the tested strains was carried out by acid methanolysis of the whole biomass in 0.4 ml of 1.2 N HCl in methanol by heating to 80°C for 1 h. The resulting fatty acid methyl esters were extracted twice with 0.2 ml of hexane and processed on a computer-assisted Microbial Identification System (MIS) (Microbial ID Inc., Newark, Del.) with a Hewlett Packard 5890A gas chromatograph and Hewlett Packard 7673A automatic sampler. The parameters of chromatography were chosen as recommended in the operational manual of the MIS [29, 30]. Gas chromatography-mass spectrometry (GC-MS) analysis was performed using an Agilent Technologies AT-5975 GC-MS system equipped with a cross-linked methyl silicone capillary column HP-5 ms. The oven temperature was 135°C for 2 min and then programmed up to 320°C at 7 degrees/min. Next, 1–2 *μ*l of the derivatized sample was injected into the gas chromatograph at 280°C. Fatty acids and other lipid components were ionized by electron impact at 70 eV after separation in the GC column and analyzed in the scan mode. The quadrupole mass spectrometer had a resolution of 0.5 mass units over the whole mass range of 2–950 amu. The sensitivity of the GC-MS system was 0.01 ng of methyl stearate. Each substance was confirmed by its mass spectrum and a NIST mass spectral database library search.

## 3. Results

### 3.1. Synthesis of AHL Signaling Molecules by the *S. proteamaculans* 94 Strain

For detailed determination of AHL production, LC-MS/MS analysis was used. It was shown that strain 94 synthesized several types of AHLs, the amount of which significantly differed (Table 3). *S. proteamaculans* 94 cells synthesized and secreted OHHL and HHHL in the largest quantities, whereas the amounts of produced HHL, OHL, and BHL were much smaller. Trace amounts of ODHL, DDHL, and OOHL were also found in cell-free supernatants.

### 3.2. Nucleotide Sequence and Organization of the *sprI* and *sprR* Genes in *S. proteamaculans* 94

The nucleotide sequences of the *sprI* and *sprR* genes were determined and annotated (GenBank, JX901285). The *sprI* and *sprR* genes are 633 and 750 bp in size, respectively. A comparison of the nucleotide sequences of the *S. proteamaculans* 94 genes *sprI* and *sprR* to those of the corresponding genes of other bacteria revealed high similarity of the QS genes in *Serratia* species: the *S. proteamaculans* strain B5a (83 and 88% identity of the *sprI* and *sprR* genes, respectively, AY040209.1), the *Serratia plymuthica* RVH1 strain (83 and 87% identity, AY394723.2), HRO-C48 strain (83 and 87% identity, AY841161.1), and G3 strain (83 and 87% identity, FJ919305.1); the *Serratia liquefaciens* HUMV-21 strain (79 and 85% identity, CP011303.1) and FDAARGOS_125 strain (79 and 85% identity, CP014017.1); and the *Serratia marcescens* H30 strain (77 and 81% identity, EU570248.1 and EU570249.1). High similarity of QS genes was also found with the corresponding genes of other genera of *Enterobacteriaceae*: the *Yersinia enterocolitica* LC20 (76 and 81% identity, CP007450.1); the *Erwinia* sp. EM595 (72 and 75%, LN907828.1); *Pantoea ananatis* strain LMG2665 (72 and 75%, KM249358.1); and *Hafnia* sp. CBA7124 (73 and 73%, AP017469.1).

**Figure 1:**
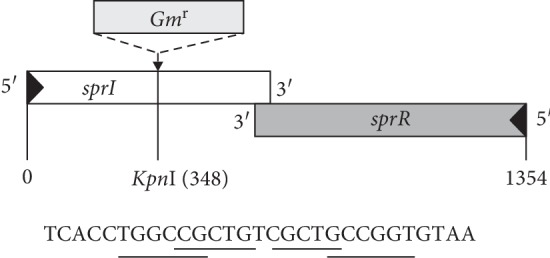
Organization of *S. proteamaculans* 94 QS genes and location of the Gm^r^ cassette insertion in the *sprI* gene. The arrows indicate the direction of transcription. The bottom part of the figure shows the nucleotide sequence of the region of overlapping of *spr*I and *spr*R genes. The repeats presumable are underlined.

The *sprI* and *sprR* genes of *S. proteamaculans* 94 partially overlapped at their 3′-end regions and were transcribed convergently from opposite DNA strands. The area of overlap is 23 bp long and rich in GC-bases (18 GC out of 23 bp), and it contains repeats (underlined) (Figure 1).

**Figure 2:**
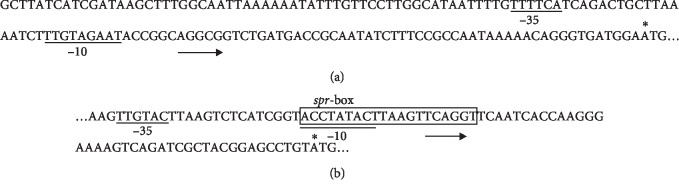
Promoter regions of the *sprI* (a) and *sprR* (b) genes. The sequences underlined are presumable −10 and −35 sites, and the initiation codon is indicated by an asterisk. The selected sequence is a presumable *spr*-box. The arrows indicate the direction of transcription.

We have analyzed the sequence of a DNA fragment carrying the QS system genes *sprI* and *sprR* and adjacent regions with the help of promoter analysis programs (http://molbiol-tools.ca, PPP—Prokaryotic Promoter Prediction). As a result, we found the most likely sequences of −10 and −35 sites of the promoters of the *sprI* and *sprR* genes in *S. proteamaculans* 94 (Figure 2).

A site similar to a *lux*-box of *V. fischeri* [31] was detected upstream of the *sprR* gene (an *spr*-box) (Figure 2(b)). The sequence of the *spr-*box contains a probable −10 site in the promoter of the SprR protein gene, binding of this protein to the *spr*-box could presumably inhibit initiation of transcription of the gene encoding the SprR protein.

### 3.3. Construction of a Mutant with Knocked-Out *sprI* Gene

To determine how the absence of synthesis of AHL signaling molecules affects the properties of *S. proteamaculans* 94 cells, we constructed a mutant bearing an inactivated *sprI* gene. Figure 1 shows the location of the Gm-r cassette insertion in this gene. Using the biosensors *C. violaceum* CV026 and *A. tumefaciens* NT1/pZLR4, we showed that the *S. proteamaculans sprI*::Gm mutant with the inactivated AHL synthase gene did not produce AHL.

To test whether AHL synthesis in *sprI* mutant cells would be restored by introduction of cloned QS genes into the cells, we used a pUCP26-*splIR* plasmid containing the QS system genes of the closely related bacterium *S. plymuthica* HRO-C48 for a complementation experiment [23]. As indicated above, the *sprI* and *splI* genes of the *S. proteamaculans* 94 and *S. plymuthica* HRO-C48 strains are highly similar (83 and 87% identity, respectively). The pUCP26-*splIR* plasmid was transferred by electroporation into *S. proteamaculans* 94 and the *sprI*::Gm mutant. The obtained transformants were selected for resistance to tetracycline (40 µg/ml). The presence of the pUCP26-*splIR* plasmid in transformants was tested by PCR using the TET-F/TET-R primers to show the presence of the tetracycline resistance gene of the vector plasmid and the SplI–F/SplI–R primers to confirm the presence of the *splI*/*splR* genes [23]. Introduction of the pUCP26-*splIR* plasmid into *sprI*::Gm mutant cells restored the synthesis of AHL (Table 4).

### 3.4. Effect of Inactivation of the *sprI* Gene on the Properties of *S. proteamaculans* 94

We studied the influence of inactivation of the *sprI* gene on the activities of some extracellular enzymes synthesized by strain 94. It was shown that the proteolytic activity in the mutant strain *sprI*::Gm was reduced (Table 4). The mutant strain lacked the chitinolytic activity of the parent strain (Table 4). The original and mutant strains grown on blood agar plates showed almost identical transparent small zones of hemolysis with radii of 1–2 mm; lipase activity (turbid zones around colonies of the parent and mutant strains) was similar (Table 4). Earlier, we showed that *S. proteamaculans* 94 produced volatile compounds, including VOCs (with dimethyl disulfide as the main VOC) that suppressed the growth of bacteria and fungi [27]. In this work, we found that inactivation of the *sprI* gene reduced or completely inhibited the growth suppression of two fungi, *R. solani* and *H. sativum*, induced by volatile substances (Table 4). We also tested the effect of the *sprI* gene inactivation on the ability of cells to migrate by swimming. It was shown that swimming zones were absent in the case of the *sprI*::Gm mutant (Table 4).

Introduction of the pUCP26-*splIR* plasmid into *sprI*::Gm mutant cells restored the characteristics of the parent strain for properties studied (Table 4).

### 3.5. Analysis of Fatty Acid Composition in *S. proteamaculans* 94 and the *sprI*::Gm Mutant

Saturated, unsaturated, and hydroxy acids were found in samples of *S. proteamaculans* 94 and the *sprI*::Gm mutant (Table 5). In total, 22 fatty acids with a chain of 12 to 20 carbon atoms were identified. The main saturated acids in *S. proteamaculans* 94 were hexadecanoic, octadecanoic and tetradecanoic acids, constituting approximately 37%, 11%, and 2.7% of the total amount of fatty acids in this strain, respectively. The dominant unsaturated acids were 9-octadecenoic, 11-octadecenoic, and 9-hexadecenoic (33%, 4.6%, and 3.4%, respectively). The unsaturation coefficient of fatty acids (the ratio of total unsaturated fatty acids to total saturated fatty acids) in the strain *S. proteamaculans* 94 was 0.83.

The *sprI*::Gm mutant of strain 94 was characterized by significant changes in the amount of fatty acids compared to that of the wild-type strain (for example, nos. 8, 10, 14, and 16) (Table 5). Fatty acids in the mutant strain were characterized by lower unsaturation compared with those in the parent *S. proteamaculans* 94 strain. The unsaturation coefficient of fatty acids of the *sprI*::Gm mutant was 0.63. The composition of fatty acids of the mutant strain differed from those of the wild-type strain by a sharply increased content of cyclopropane-heptadecanoic and 3-hydroxy-tetradecanoic acids (∼23- and 10-fold for the *sprI*::Gm mutant, respectively). Several fatty acids (nos. 7, 11, 12, 13, and 21) were absent in the mutant strain in contrast to *S. proteamaculans* 94. Three fatty acids (nos. 2, 3, and 5) absent in strain 94 were found in the *sprI*::Gm mutant strain in an amount less than 1%. Thus, the data above show that inactivation of the *sprI* gene in *S. proteamaculans* 94 significantly affected the composition of fatty acids.

## 4. Discussion

Intensive studies of QS regulation systems carried out in the recent years have shown that this type of regulation is widespread in bacteria of different taxonomic groups. AHL signals synthesized by different *Serratia* species are represented by mainly BHL, HHL, OHL, and OHHL and rarely also by *N*-hydroxy-acyl-*L*-homoserine lactones [8, 25, 32]. Our work is devoted to the study of the QS system in one of the representatives of the genus *Serratia*, namely, in the *S. proteamaculans* 94 strain isolated from spoiled refrigerated meat. QS systems in *S. proteamaculans* have been little studied. As far as we know, the only exception is the B5a strain, which produces OHHL as the main AHL [11]. A preliminary analysis of AHL production in *S. proteamaculans* 94 performed with the *C. violaceum* CV026 and *A. tumefaciens* NT1/pZLR4 biosensors demonstrated AHL synthesis in the *S. proteamaculans* 94 strain [16]. Quantitative mass spectrometry analysis showed that the *S*. *proteamaculans* strain synthesized five types of AHLs after 24 h of growth, among which OHHL and HHHL were predominant. The amounts of synthesized AHLs were on the same order as, for example, those in *Pseudomonas aeruginosa.* At least two concentration levels were observed in culture supernatants of this bacterium: AHLs at a relatively high content (5–15 *μ*M) and those occurring at a lower content (<0.2 *μ*M) [33].

Cloning and sequencing of the *S. proteamaculans* 94 AHL synthase *sprI* gene and the *sprR* gene encoding a regulatory receptor protein revealed that these genes are convergently transcribed and overlap in their terminal areas. Such QS system gene expression is different from that of the classical LuxI-LuxR QS model of *V. fischeri*, where the *luxI* and *luxR* genes are transcribed divergently [34]. The same difference was found in other members of the *Enterobacteriaceae* family, including species of *Serratia*, *Erwinia,* and *Pantoea* [5, 11, 35–37]. The area of overlap of the *sprI* and *sprR* genes is highly similar to the corresponding regions in other *Serratia* species: there is up to 100% identity with the area of overlap of these genes in *S*. *proteamaculans* B5a [11], *S. plymuthica* HRO-C48 [23], G3 [32], and RVH1 [38], and somewhat less similarity in other representatives of *Serratia*, for example, 70% sequence identity in *S. marcescens* [39]. However, the functional role of such overlapping QS genes is currently unclear.

A characteristic feature of the *S. proteamaculans* 94 QS system is the presence of the *spr*-box, which is similar to the *lux*-box of *V. fischeri*, in the promoter region of the *sprR* gene. A *lux*-box of *V. fischeri* is a 20 bp inverted repeat sequence of DNA that binds the QS regulatory receptor LuxR protein [31]. The *spr*-box in *S. proteamaculans* 94 overlaps with a tentative −10 site in the *sprR* gene promoter. Therefore, the binding of the SprR protein to the *spr*-box could apparently inhibit transcription of the *sprR* gene, preventing the binding of RNA polymerase. This assumption is in good agreement with available published data on transcription regulation of the *esaR* gene of *P. stewartii*, which encodes the EsaR protein, an analog of LuxR [5, 6]. The ExpR protein of *E. chrysanthemi* also represses transcription of its gene through interaction with the *lux-*box in the absence of AHLs, the transcription being derepressed in the presence of micromolar amounts of AHLs [37]. *Lux*-like-boxes were also found in other species of *Serratia*, for example, in *S. marcescens* SS-1 and *S. plymuthica* RVH1, upstream of the *spnR* and *splR* genes, respectively [37, 38].

The important role of AHL-dependent QS systems in regulation of cellular processes has been demonstrated for various species of *Serratia*. These QS systems participate in the control of the production of extracellular enzymes and some antibiotics, as well as in biofilm formation and motility [11, 23, 38, 40]. A proteomic analysis of the *S. proteamaculans* B5a strain showed QS-controlled expression of 39 cellular proteins, including the *lipB*-encoded secretion system and several lytic enzymes [11].

To study the role of QS in the regulation of cellular processes related to various aspects of metabolism in *S. proteamaculans* 94, an *sprI* mutant was constructed using gene-replacement mutagenesis. Inactivation of the *sprI* gene resulted in a decrease in extracellular proteolytic and chitinolytic activity but did not affect lipase and hemolytic activity. A mutation in the *sprI* gene also affected swimming motility of cells and antifungal activity related to the action of volatiles emitted by the parental strain. It is known that many strains of the genus *Serratia* have antifungal activity based on production of antibiotics and other antimicrobial substances, VOCs among them [8, 23, 25, 41, 42]. We showed previously that the volatiles of the strain *S. proteamaculans* 94 inhibited the mycelium growth of several phytopathogenic fungi [27]. Here, it was found that inactivation of the *sprI* gene reduced the inhibitory effect of *S. proteamaculans* 94 volatile compounds on the phytopathogenic fungi *R. solani* and *H. sativum*, suggesting that this gene is involved in the regulation of the synthesis of these substances in strain 94. Volatile compound activity may be involved in the competition of *S. proteamaculans* with other microorganisms in the rhizosphere and soil.

It is worth noticing that previously we have inactivated *luxS* gene, that allows the synthesis of another type of signaling molecules, AI-2, in strain 94, and investigated in this mutant almost the same phenotypes as those investigated here [16], with the exception of fatty acid synthesis. The comparison of these previously published data and our current data indicates that the deficiency in AHLs and AI-2 signaling molecules (or *luxS* gene inactivation) could lead to the very similar mutant phenotype. The results that are presented here may indicate that both AHLs and AI-2 systems in strain *S. proteamaculans* 94 could be involved (separately or in coordination) in QS control of the same physiological and metabolic processes.

The composition of cellular fatty acids is a stable genetic trait that is maintained in many generations of bacteria and used for their identification [43]. The ratio of saturated and unsaturated fatty acids affects physical properties of bacterial membranes and the degree of their fluidity and has a very significant effect on the activity of membrane-bound enzymes, permeability of membranes, and ability of cells to grow at low temperatures [44, 45]. Recently, it was shown that monounsaturated chain fatty acids and palmitoleic and myristoleic acids prevented QS-dependent biofilm development and drastically reduced motility in the nosocomial pathogen *Acinetobacter baumannii* [46]. The authors highlighted that these fatty acids decrease the expression of the regulator gene *abaR* belonging to the LuxIR-type QS communication system AbaIR and consequently reduce AHL synthesis. Essentially, this effect can be countered by the addition of exogenous AHLs. Moreover, oxygenated unsaturated fatty acids, known as oxylipins, produced by *P. aeruginosa*, function as autoinducers of a novel QS system [47]. The discovery of this oxylipin-dependent QS system reveals that prokaryote-derived oxylipins also mediate cell-to-cell communication in bacteria. Here, we have shown that the composition of fatty acids in *S. proteamaculans* 94 mutant cells with an inactivated QS gene *sprI* was significantly different from fatty acid composition of the original strain. This allows us to assume that the deficiency in AHL synthesis bidirectionally affects the synthesis of various fatty acids.

## 5. Conclusions

In this work, we have studied an AHL-mediated QS system in the psychrotrophic bacterium *Serratia proteamaculans* strain 94 isolated from spoiled meat. We have shown that this strain produced several types of AHLs, with OHHL and HHHL as the predominant signals. *luxIR*-like genes of the strain 94 QS system (*sprI* and *sprR*) were cloned and sequenced, and the organization of these genes was investigated. The *S. proteamaculans* 94 mutant with an inactivated *sprI* gene was found to be deficient in several physiological and metabolic traits, including AHL production, extracellular proteolytic and chitinolytic activities, swimming motility, and fungal mycelium growth suppression related to production of volatile compounds. All these phenotypes were restored to the parental strain level by complementation with similar wild-type *luxIR* genes cloned from another strain of *Serratia*. Moreover, inactivation of the *sprI* gene led to changes in the fatty acid composition detected in the wild-type strain, indicating involvement of QS regulation also in this basic metabolic process in strain 94. The obtained data provide new information about bacteria that are able to spoil various dairy, meat, or fish products, which may help to control food spoilage, for instance, by disrupting QS networks related with this undesirable activity of *S. proteamaculans*.

## Figures and Tables

**Table 1:** Strains and plasmids used in this study.

Strains or plasmids	Characteristics	Source or reference
Strains		
*Serratia proteamaculans* 94	Isolated from spoiled refrigerated meat	Demidyuk et al. [10]
*S. proteamaculans* 94 *rif*-r	*rif*-r spontaneous mutant	Zaitseva et al. [16]
*Escherichia coli* S17-1 (*λ*-*pir*)	*thi pro hsdR hsdM recA rpsL* RP4-2 (Tc^r^::Mu) (Km^r^::Tn7) (*λ*-*pir*)	Simon et al. [17]
*E. coli* TG-1	K-12 *supE thi-1* Δ(*lac-proAB*) Δ(*mcrB-hsdSM*)5, (r_K_^−^m_K_^−^)	StrataGene
	F′ [*tra D36 proAB*^*+*^*lacI*^*q*^*lac* ZΔM15]	
*E. coli* XL-1 blue	*recA1 endA1 gyrA96 thi1 hsdR17 supE44 relA1 lac*	Stratagene
*Chromobacterium violaceum* CV026	Violacein production-based AHL bioreporter, Km-r	McClean et al. [18]
*Agrobacterium tumefaciens* NT1/pZLR4	pZLR4 *traG*::*lacZ β*-galactosidase induction-based AHL bioreporter, Gm^r^ Cb^r^	Shaw et al. [19]
*S. proteamaculans* 94 *rif*-r *sprI*::Gm	*sprI*::Gm gene replacement mutant of strain 94 *rif*-r	This study

Plasmids		
p34S-Gm	Source of Gm^r^-cassette	Dennis and Zylstra [20]
pEX18Tc	Gene replacement vector, Tet^r^ oriT *sacB*	Hoang et al. [21]
pAL-TA	Vector for cloning of PCR-products, Ap^r^ ori pUC P_lac_	Eurogen
pACYC184	Vector for cloning, ori p15 A, Tet^r^ Cam^r^	Sambrook and Russel [22]
pUCP26-*splIR*	*splI* and *splR* genes cloned in pUCP26-host-range vector, Tc^r^	Liu et al. [23]

**Table 2:** Primers used in this study.

Primer	Sequence (5′ ⟶ 3′)
deg SprI-F	5′-ATGCTTGAA(C,T)T(A,G)TTTGA(C,T)GT(C,T)AG
deg SprI-R	5'-(G,C)GGCCAGGT(A,G)ATAAC(T,G,C)GA
SprI-F	5′-ATGCTTGAATTATTTGACGTCAG+
SprI-R	5′-GCTGGAACTTATTACACCGG+
SprR-F	5′-GAGCCTGTATGTTTTCCATC+
SprR-R	5′- CAACTTCCGCCATCACCTG+
pACYC-F	5′-CAAATGTAGCACCTGAAGTC+
pACYC-R	5′- CGATGCGTCCGGCGTAGAG+
GM-F	5′-GGCTCAAGTATGGGCATCATT+
GM-R	5′-GGCGGTACTTGGGTCGATA+
М13-F	5′-GTAAAACGACGGCCAGT+
М13-R	5′-CAGGAAACAGCTATGAC+
TET-F	5′-CGAACGCCAGCAAGACGTAG
TET-R	5′-CTGCTCGCTTCGCTACTTGG

**Table 3:** Amount of AHLs detected in *S. proteamaculans* 94 culture supernatants by LC-MS/MS analysis.

AHL	BHL	HHL	OHHL	HHHL	HOHL	OHL	ODHL	OdDHL	DDHL	OOHL
ng/ml^*∗*^	**12.5**	**41**	**>2500**	**>2000**	nd	**5.5**	**2**	nd	**2**	Traces
*μ*M^*∗∗*^	**<0.1**	**0.2**	**>11.7**	**>9.3**	nd	**<0.1**	**<0.01**	nd	**<0.01**	**<0.001**

nd, not detected. Concentration of AHLs in EtAc extracts, which were prepared from supernatants of strain *S. proteamaculans* 94 strain, was measured (mass^*∗*^ and molar^*∗∗*^ concentrations). Analysis was performed after 24 hours of bacterial growth.

**Table 4:** Effect of mutation *spr*I:Gm and introduction of pUCP26-*spl*IR plasmid into the mutant on the properties of cells^*∗*^.

Properties	*S. proteamaculans* 94	*sprI:*Gm	*sprI*:Gm/pUCP26-*splIR*
AHL production^*∗∗*^	+	−	+
Extracellular proteolytic activity^a^	5.1 ± 2.0	1.2 ± 0.0	4.8 ± 1.5
Chitinolytic activity^b^	4.6 ± 1.5	0	4.2 ± 1.0
Lipolytic activity^c^	5.4 ± 1.3	4.6 ± 1.5	4.1 ± 2.0
Hemolytic activity^d^	2.0 ± 1.0	1.6 ± 0.6	1.9 ± 0.4
Effect of VOCs on *R. solani* growth^e^	7.7 ± 1.5	19.6 ± 3.0	8.4 ± 1.0
Effect of VOCs on *H. sativum* growth^e^	8.6 ± 2.5	21.4 ± 2.0	7.5 ± 1.5
Swimming^f^	33.0 ± 2.5	0	24.0 ± 2.0

^*∗*^The results of four independent experiments are expressed as the mean ± standard deviation (SD). ^*∗∗*^Assay of AHL production was performed with the *C. violaceum* CV026 and *A. tumefaciens* NT1/pZLR4 biosensors. ^a^Radius of the zone of casein hydrolysis, mm. ^b^Radius of the zone of hydrolysis of chitin, mm. ^c^Radius of the turbid zones around the colonies, mm. ^d^Radius of the zone of hemolysis, mm. ^e^Growth of mycelium measured as distance in mm between the block of fungus and the border of its mycelium (action of *S. proteamaculans* 94 volatile compounds). In the absence of *S. proteamaculans* 94, the growth of fungal mycelium was 20–24 mm. ^f^Diameter of the bacterial growth of the strains around a 2 *μ*l drop of culture grown for 36 h at 30°C.

**Table 5:** Fatty acid composition in the strains *S. proteamaculans* 94 (A) and *S. proteamaculans spr*I::Gm (B).

No.	RT^*∗*^	Fatty acid	Amount of fatty acids in percent of the sum of areas of all chromatographic peaks	
			A	B
1	6.983	Dodecanoic	0.5 ± 0.1	0.9 ± 0.1
2	9.495	9-Tetradecenoic	0	0.2 ± 0.1
3	9.578	11-Tetradecenoic	0	0.3 ± 0.1
4	9.873	Tetradecanoic	2.7 ± 0.3	4.9 ± 0.4
5	10.120	2-Hydroxy-dodecanoic	0	0.2 ± 0.1
6	11.305	Pentadecanoic	0.6 ± 0.1	0.6 ± 0.1
7	12.379	7-Hexadecenoic	0.4 ± 0.1	0
8	12.449	9-Hexadecenoic	3.4 ± 1.2	27.6 ± 2.4
9	12.797	Hexadecanoic	37.2 ± 1.7	34.6 ± 0.8
10	12.839	3-Hydroxy-tetradecanoic	0.6 ± 0.1	6.4 ± 0.5
11	13.570	Isoheptadecanoic	0.2 ± 0.1	0
12	13.694	Anteiso heptadecanoic	0.3 ± 0.1	0
13	13.764	Heptadecenoic	0.2 ± 0.0	0
14	13.888	Cyclopropane heptadecanoic	0.2 ± 0.1	5.5 ± 0.3
15	14.071	Heptadecanoic	0.8 ± 0.1	3.3 ± 0.2
16	15.144	9-Octadecenoic	33.2 ± 1.3	9.4 ± 2.1
17	15.186	11-Octadecenoic	4.6 ± 0.2	0.3 ± 0.1
18	15.427	Octadecanoic	11.0 ± 0.3	5.1 ± 0.2
19	16.170	Octadecadienoic, conjugated	1.3 ± 0.1	0.5 ± 0.1
20	17.556	9-Eicosenoic	1.5 ± 0.2	0.3 ± 0.0
21	17.597	11-Eicosenoic	0.9 ± 0.2	0
22	17.845	Eicosanoic	0.5 ± 0.1	0.2 ± 0.1
Coefficient of unsaturation of fatty acids			0.83	0.63

The numbers in the table are mean ± standard deviation (SD) of the analysis of the two independently grown cultures of each variant. RT: chromatographic retention time of fatty acids.

## Data Availability

The data used to support the findings of this study are available from the corresponding author upon request.

## Abbreviations

QS: Quorum sensing
AHL: *N*-Acyl-*L*-homoserine lactone
BHL: *N*-Butanoyl-*L*-homoserine lactone
HHL: *N*-Hexanoyl-*L*-homoserine lactone
OHHL: *N*-(3-Oxo-hexanoyl)-*L*-homoserine lactone
HHHL: *N*-(3-Hydroxy-hexanoyl)-*L*-homoserine lactone
HOHL: *N*-(3-Hydroxy-octanoyl)-*L*-homoserine lactone
OHL: *N*-Octanoyl-*L*-homoserine lactone
ODHL: *N*-(3-Oxo-decanoyl)-*L*-homoserine lactone
OdDHL: *N*-(3-Oxo-dodecanoyl)-*L*-homoserine lactone
DDHL: *N*-Dodecanoyl-*L*-homoserine lactone
OOHL: *N*-(3-Oxo-octanoyl)-*L*-homoserine lactone
EtAc: Ethyl acetate
AI-2: Autoinducer 2
VOC: Volatile organic compound.
